# Hepatic NK cell-mediated hypersensitivity to ConA-induced liver injury in mouse liver expressing hepatitis C virus polyprotein

**DOI:** 10.18632/oncotarget.11052

**Published:** 2016-08-04

**Authors:** Qiuxia Fu, Shaoduo Yan, Licui Wang, Xiangguo Duan, Lei Wang, Yue Wang, Tao Wu, Xiaohui Wang, Jie An, Yulong Zhang, Qianqian Zhou, Linsheng Zhan

**Affiliations:** ^1^ Beijing Institute of Transfusion Medicine, Beijing Key Laboratory of Blood Safety and Supply Technologies, Beijing, China; ^2^ Surgical Laboratory of General Hospital, Ningxia Medical University, Yinchuan, China; ^3^ Blood Transfusion Department, General Hospital of Beijing Military Area Command of PLA, Beijing, China

**Keywords:** HCV, codon-optimized φC31 integrase, NK cells, liver injury

## Abstract

The role of hepatic NK cells in the pathogenesis of HCV-associated hepatic failure is incompletely understood. In this study, we investigated the effect of HCV on ConA-induced immunological hepatic injury and the influence of HCV on hepatic NK cell activation in the liver after ConA administration. An immunocompetent HCV mouse model that encodes the entire viral polyprotein in a liver-specific manner based on hydrodynamic injection and φC31o integrase was used to study the role of hepatic NK cells. Interestingly, the frequency of hepatic NK cells was reduced in HCV mice, whereas the levels of other intrahepatic lymphocytes remained unaltered. Next, we investigated whether the reduction in NK cells within HCV mouse livers might elicit an effect on immune-mediated liver injury. HCV mice were subjected to acute liver injury models upon ConA administration. We observed that HCV mice developed more severe ConA-induced immune-mediated hepatitis, which was dependent on the accumulated intrahepatic NK cells. Our results indicated that after the administration of ConA, NK cells not only mediated liver injury through the production of immunoregulatory cytokines (IFN-γ, TNF-α and perforin) with direct antiviral activity, but they also killed target cells directly through the TRAIL/DR5 and NKG2D/NKG2D ligand signaling pathway in HCV mice. Our findings suggest a critical role for NK cells in oversensitive liver injury during chronic HCV infection.

## INTRODUCTION

Hepatitis C virus (HCV) is a major cause of chronic hepatitis that can lead to cirrhosis and hepatocellular carcinoma [1–2]. A certain percentage of HCV-infected patients are susceptible to biochemical and histological activation of hepatocyte injury, which is often complicated by the programmed responses of inflammation and regeneration and is a serious risk for the development of liver cirrhosis and hepatocellular carcinoma [3–5]. A clear understanding of the susceptibility to hepatocyte injury in chronic HCV patients will eventually facilitate the development of novel treatment strategies for HCV liver disease.

Because HCV itself is not cytopathic, liver damage in chronic hepatitis C is commonly attributed to immune-mediated mechanisms [6–8]. The innate immune system provides the first line of defense in antiviral responses and activates adaptive immune responses. NK cells have been considered the most important effectors of the initial antiviral innate immune system. Unlike T and B cells, NK cells efficiently exert their effector functions, classically without the necessity of prior sensitization and mainly through direct cytotoxicity and the production of various cytokines. In addition, NK cells have been identified as adaptive immune response regulators via cross-talk with dendritic cells and T cells. Given the central role of NK cells in both direct and indirect modulation of the immune system, NK cells have been shown to be crucial in defense against HCV and in the pathogenesis of HCV-induced hepatitis [9–12].

As an organ with predominant innate immunity, the liver harbors distinct resident populations of NK cells, which play critical roles in liver defense against pathogenic microbes and tumors [13–15]. Importantly, the phenotype and function of hepatic NK cells are altered during pathogen infection. In different liver microenvironments, hepatic NK cells exhibit unique NK cell repertoires and cytokine profiles. However, the role of hepatic NK cells in the pathogenesis of HCV-associated hepatic failure is not well understood. Further studies are needed to define these questions.

Concanavalin A (ConA)-induced hepatitis is a well-characterized murine model for studying the mechanisms and therapy of immune-mediated hepatotoxicity. It is characterized by massive hepatocellular degeneration and lymphoid infiltration in the liver [16–17]. Previous studies have shown that ConA administration can activate innate immune cells, including hepatic NK cells and NKT cells [18]. In addition, NK cells have been shown to be involved in the sensitive liver injury process triggered by the ConA in HBV transgenic mice [19]. However, the correlation between HCV and ConA-induced immunological hepatic injury, particularly the influence of HCV on hepatic NK cell activation in the liver after ConA administration, remains obscure.

Thus, this report focuses on the changes in NK cells in the liver induced by ConA injection using an immunocompetent HCV mouse model that encodes the entire viral polyprotein in a liver-specific fashion. We showed that HCV mice were overly sensitive to ConA-induced liver injury and that hepatic NK cells were the pivotal mediators in ConA-induced liver damage in HCV mice via cytokine production and direct cytotoxicity against hepatocytes.

## RESULTS

### Stable gene expression of HCV and the reporter gene in mouse hepatocytes mediated by φC31 integrase through hydrodynamic injection

**Figure 1:**
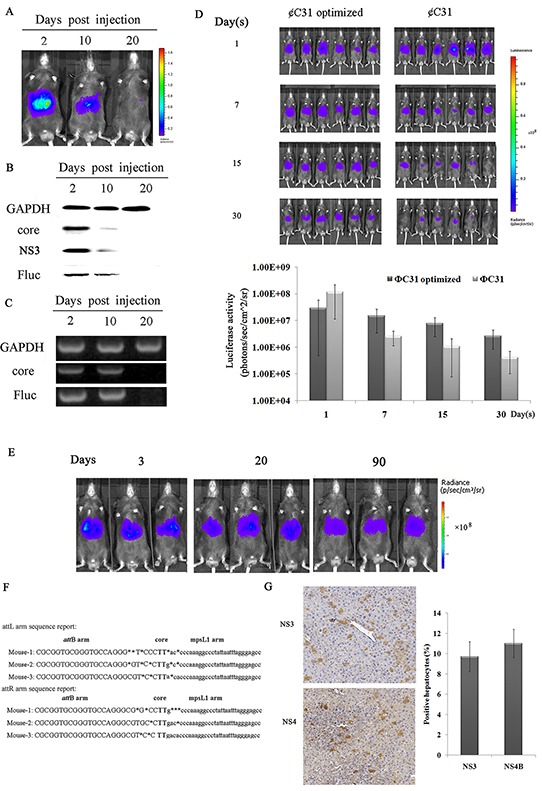
A mouse model that can express both HCV and luciferase genes in hepatic tissues **A–C**. Transient expression of HCV and the reporter gene in the mouse liver analyzed by *in vivo* imaging, RT-PCR and western blotting. (A) Analysis of mice injected with p*att*B-HCV-Fluc by *in vivo* imaging at 2, 10 and 20 days post-injection. The results are representative of three experiments (n=3). (B) Liver extracts from p*att*B-HCV-Fluc-injected mice collected at the indicated time points were separated by SDS-PAGE, and immunoblots were probed with anti-core, anti-NS3 and anti-luciferase antibodies. GAPDH served as a loading control. Representative photographs of specific bands from three independent experiments are shown. (C) RT-PCR analysis of the expression of HCV and luciferase in murine livers at the indicated time points. Representative photographs of specific bands from three individual experiments are shown. **D–G**. Sustained HCV expression in murine livers. (D) Analysis of φC31 and φC31o integrase activity in murine livers. Bioluminescence imaging of mice co-injected with p*att*B-HCV-Fluc plus pCMV-int or Pphic31φ on days 1, 7, 15 and 30 post-injection and the corresponding bioluminescence intensities. The results are representative of two experiments (n=6). (E) Real-time bioluminescence imaging of mice co-injected with Pphic31φ plus p*att*B-HCV-Fluc on days 3, 20, and 90 post-injection. Representative images of three experiments are shown (five to ten mice per group). (F) An alignment of the amplified *att*L and *att*R vector-genome junctions from hepatic genomic DNA of mice that received Pphic31φ and the *att*B donor (n=3). (G) Immunohistochemical analysis of the expression of HCV in murine livers on day 90 post-injection. One of three experiments is shown.

To study the role of hepatic NK cells in the pathogenesis of HCV-associated hepatic failure, we developed a novel system (Supplementary Figure S1) based on a pCIneo vector that could express both the reporter gene luciferase and HCV polyprotein synchronously off the AAT enhancer/promoter. To generate a mouse model capable of expressing both the HCV and luciferase genes in hepatic tissues, 10μg of p*att*B-HCV-Fluc was delivered into each mouse through a hydrodynamic injection method, as described in materials and methods. To confirm the expression of HCV proteins, the mice were analyzed by *in vivo* imaging, RT-PCR and western blotting (as detected by anti-luciferase, anti-core, anti-NS3 and anti-GAPDH) at 2, 10 and 20 days post-injection. The results revealed that hydrodynamic injection of p*att*B-HCV-Fluc induced synchronous expression of HCV and the reporter gene (Figure 1A,B and 1C) *in vivo*. Thus, the use of luciferase as a reporter strategy represents a platform for near real-time detection of HCV expression in living mice. Furthermore, the expression of HCV and luciferase became undetectable at day 20, indicating that the gene delivery procedure only resulted in transient transfection of the hepatic tissues. Additionally, the effect of the hydrodynamic injection of pattB-HCV-Fluc on liver pathology was investigated. Histological examination of the livers revealed that liver morphology significantly changed in all of the animals injected with pattB-HCV-Fluc at 1 day after injection and gradually returned to a normal architecture within 3 days (Supplementary Figure S2A), consistent with previous findings [20]. Serum levels of alanine aminotransferase (ALT) and aspartate aminotransferase (AST) exhibited concordant trends (Supplementary Figure S2B). ALT levels significantly increased (approximately 700 IU/L) at 1 day after injection, subsided to 50-90 IU/L after 3 days, and returned to baseline levels by 6 days, suggesting an acute inflammation of the mouse liver. The same phenomenon was observed for the AST levels (Supplementary Figure S2B). Taken together, these results suggested that the hydrodynamic DNA delivery procedure elicited an acute hepatic inflammatory process.

A distinct band corresponding to the size of free NS3 is evident in Figure 1B, indicating that the HCV polyprotein was properly processed and led to the release of mature protein. Additionally, whether functional HCV protease was properly produced was verified *in vitro*. It has been previously reported that the HCV NS3/4A protease cleaves MAVS from mitochondria [21, 22]. Our results showed that in huh7 cells cotransfected with eYFP-MAVS and pattB-HCV-Fluc, eYFP-MAVS was proteolytically cleaved, as reported previously, and its localization shifted from the mitochondrial membrane to the cytoplasm (Supplementary Figure S3). Taken together, these results suggested that the HCV polyprotein was properly processed and that the functional HCV protease was properly produced.

To facilitate stable integration of p*att*B-HCV-Fluc, phage φC31 integrase was used. Previous studies have demonstrated that the φC31 integrase system represents a potential modality for genetic engineering of the murine genome [23, 24, 25]. However, it is inefficient and exhibits a low integration rate. Until recently, a mouse codon-optimized φC31integrase, namely φC31o integrase, which greatly improved the recombination efficiency in mouse embryos and ES cells, facilitated a potentially broader range of molecular manipulations [26, 27]. However, whether φC31o is functional in the liver of adult C57BL/6 mice remains unknown. To assess the recombination efficiency of φC31o in murine livers, 20μg of the φC31 integrase expression vector pCMV-int or the φC31o integrase expression vector Pphic31φ was co-transfected together with 10μg of p*att*B-HCV-Fluc into mouse livers by hydrodynamic injection. As shown in Figure 1D, all of the mice that were co-injected with φC31o integrase expression vector displayed high luciferase activity for more than 30 days, and the bioluminescence intensity in our model mice could be sustained at approximately 10^6^∼10^7^ p/s/cm^2^/sr. In striking contrast, the majority of the mice that were co-injected with the φC31 integrase expression vector displayed either no or low luciferase activity, whereas only a few exhibited high luciferase activity. This finding indicated that φC31o improved the recombination efficiency in C57BL/6 mouse livers and might represent a feasible and viable alternative method to create genetically modified laboratory mouse models.

The ΦC31o integrase was then utilized to prolong HCV expression in the liver. p*att*B-HCV-Fluc was hydrodynamically injected into mouse tail veins together with the φC31o integrase expression vector Pphic31φ. p*att*B-Fluc was similarly co-injected with Pphic31φ to as a control. The levels of luciferase, which were monitored over time, remained at high levels for more than 90 days (Figure 1E), indicating that recombination had occurred. The successrate of transfection of plasmid pattB-HCV-Fluc in the mouse genome exceeded 90% (Supplementary Figure S4).

Next, whether the stabilized expression levels were associated with the presence of the integrase was determined. One site in the mouse genome, *mps*L1, which was more frequently targeted for integration events than other sites [23, 24, 25], was investigated by nested PCR. The resulting PCR products were sequenced and aligned with the murine genomic sequence. The switch from the *att*B site to the genomic sequence near the TG core and the sequence between the genomic sequence and the *att*P site support φC31o-mediated integration at genomic pseudo-*att*P sites (Figure 1F). Furthermore, to confirm the expression rate of HCV proteins in murine liver, immunohistochemical staining for NS3 and NS4B was performed. As shown in Figure 1G, approximately 8-14% of the liver tissues exhibited immunopositivity for NS3 or NS4B expression on day 90 post-injection, similarly to most patients with chronic HCV infection [28].

### HCV expression reduces hepatic NK cell populations

**Figure 2:**
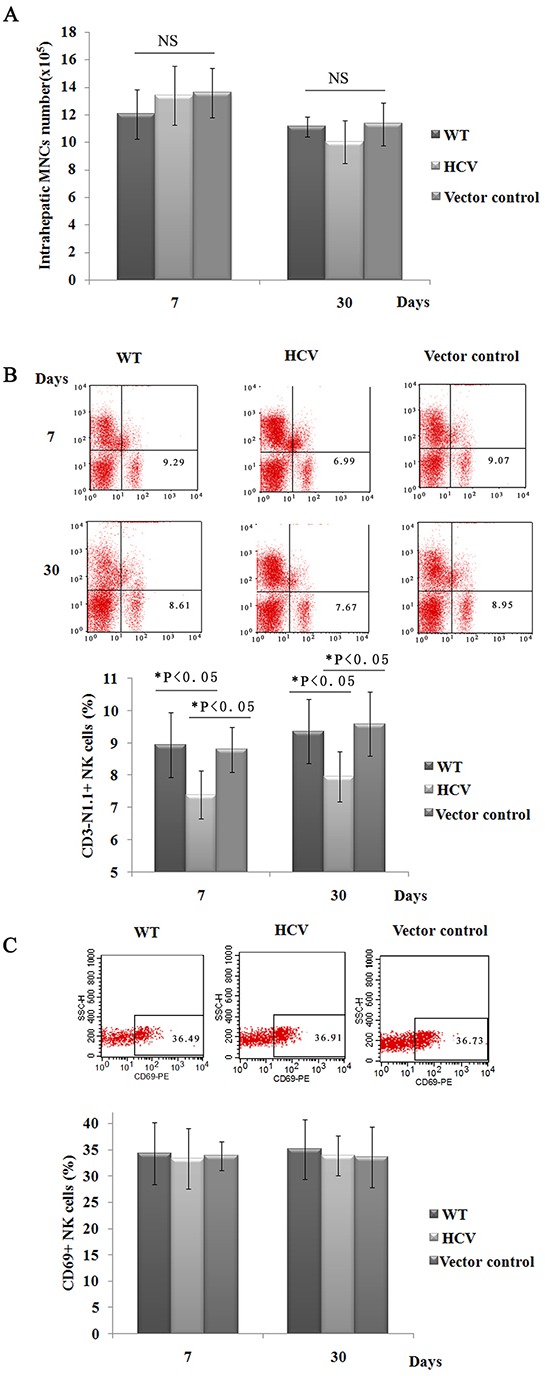
Hepatic NK cell populations are reduced in HCV mice **A**. Intrahepatic MNCs were prepared, and the total number of intrahepatic MNCs was counted at 20 days post-hydrodynamic injection. One of three experiments is shown (n=5). **B**. and **C**. Hepatic MNCs were also immunostained with FITC-conjugated anti-NK1.1, PE-Cy™5-conjugated anti-CD3e, and PE-conjugated anti-CD69. The percentages of isolated NK cells, T cells and NKT cells were determined by FACS analysis. The results shown represent one of three independent experiments. The absolute numbers of CD3- NK1.1+ cells were calculated by multiplying their respective percentages with the total liver MNC amount (B). A representative FACS analysis of the dynamic expression of CD69 on hepatic NK cells (CD3-NK1.1+, n=4). The results presented represent one of three independent experiments (C).

To determine whether sustained HCV expression in mouse liver elicits a significant effect on subsets of hepatic lymphocytes, hepatic lymphocytes were then isolated and analyzed by flow cytometry at 7 days and 30 days post-injection. The interface cells obtained between the Percoll solutions were immunostained with PerCP-conjugated anti-CD45, and the purification of intrahepatic MNCs was assessed by FACS analysis. The results showed that almost all interface cells were positive for CD45 (Supplementary Figure S5). As shown in Figure 2A, the number of intrahepatic MNCs exhibited no differences between the WT, HCV and vector control groups. The proportion of hepatic CD3-NK1.1+ NK cells in the HCV mice were significantly lower than those observed in the vector control mice (7.40% versus 9.30% at day 7, 7.96% versus 9.59% at day 30), whereas CD3+NK1.1- T cells and double-positive CD3+NK1.1+ NKT cells exhibited no difference (Supplementary Figure S6). The hepatic CD11c+ DC cells also exhibited no difference between the two strains of mice (data not shown). In addition, we analyzed the expression of CD69, an early activation marker, on NK cells. The percentages of CD69+ NK cells in the HCV group were not significantly different compared with those in the control groups (Figure 2C). These results collectively suggested that the expression of HCV genes in the murine liver dramatically decreased the presence of the hepatic NK cell population but did not impact their activation. Our findings are consistent with clinical studies that reported a significant depletion of NK cell populations in HCV patients [9–12], which likely contributes to a failure to resolve HCV infection.

Although HCV expression reduced hepatic NK cell populations, no direct cytopathic effects were found. The effect of HCV expression on liver pathology was examined over an approximately 90-day time course. Histological examination of livers (Supplementary Figure S7A) and serum levels of ALT and AST (Supplementary Figure S7B) revealed no significant histopathological changes at 90 days post-injection between the HCV and control groups, indicating that the expression of HCV elicits low levels of cytotoxicity.

### HCV mice are oversensitive to ConA-induced liver injury

**Figure 3:**
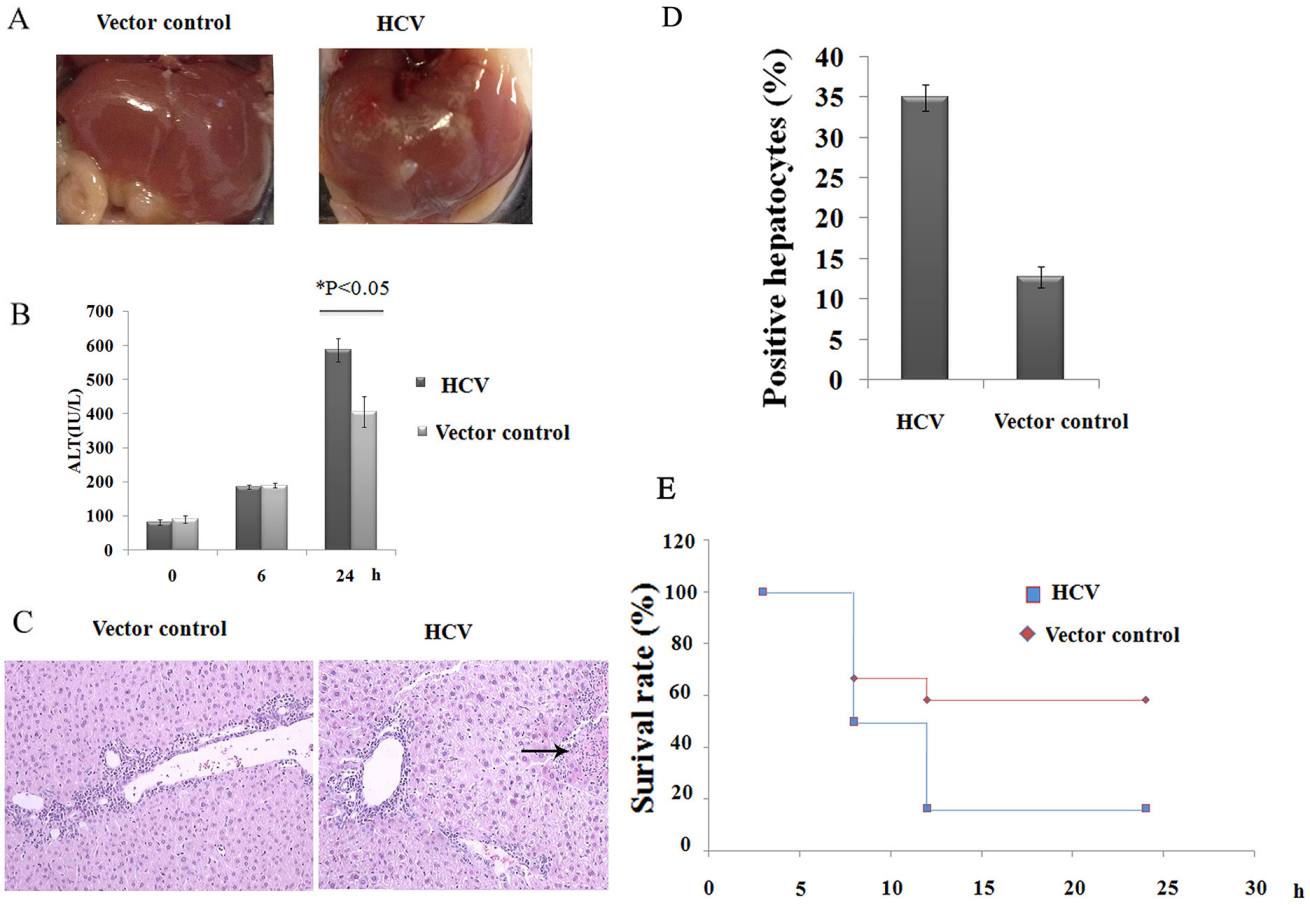
HCV mice are hypersensitive to ConA-induced hepatic injury **A**. A representative photograph of the macroscopic evaluation of livers at 24 h after ConA injection (10 μg/g body weight). **B**. A time course study of serum ALT levels at 0, 6 and 24 h post-ConA injection. The results shown represent one of three independent experiments. *P<0.05. **C**. Photomicrographs of H/E-stained liver sections and **D**. statistical analyses of the percentage of positive TUNEL-stained hepatocytes obtained from HCV and control mice at 24 h after challenge with ConA (magnification: 100×, the arrow indicates parenchymal loss). **E**. The mice were treated with a high dose of ConA (20 μg/g body weight), and the survival rates of the mice were observed after ConA injection.

An important question is whether the reduction in NK cells within HCV mouse livers might elicit an effect on immune-mediated liver injury. This mouse model was therefore used to examine the regulatory effects of HCV on immune-mediated liver injury. First, autoimmune hepatitis was induced by a systemic injection of ConA, which induces the activation of innate immune cells, including NK cells and NKT cells, as well as T cells within the liver. Initially, marked hepatic inflammation was observed upon macroscopic evaluation of the HCV mice compared with the littermate control mice (Figure 3A). Consistently, the serum levels of ALT in mice integrated with p*att*B-Fluc increased but were significantly reduced compared to those of mice integrated with p*att*B-HCV-Fluc (Figure 3B). Histological examination of liver sections documented concordant results, as evidenced by the enlarged regions of hepatocyte necrosis (Figure 3C). Immunostaining analysis revealed that nearly 31% of hepatocytes stained positive for TUNEL in the HCV group, whereas only 13% stained positive in the control group (Figure 3D). Moreover, when treated with a high dose of ConA (20 μg/g body weight), the survival rate of the HCV group was significantly reduced compared to the control group (Figure 3E). Together, these results indicated that the injection of ConA induced significantly more severe hepatocyte injury in HCV-expressing mice compared with control mice.

### Intrahepatic NK cells correlated with increased liver injury in HCV mice

**Figure 4:**
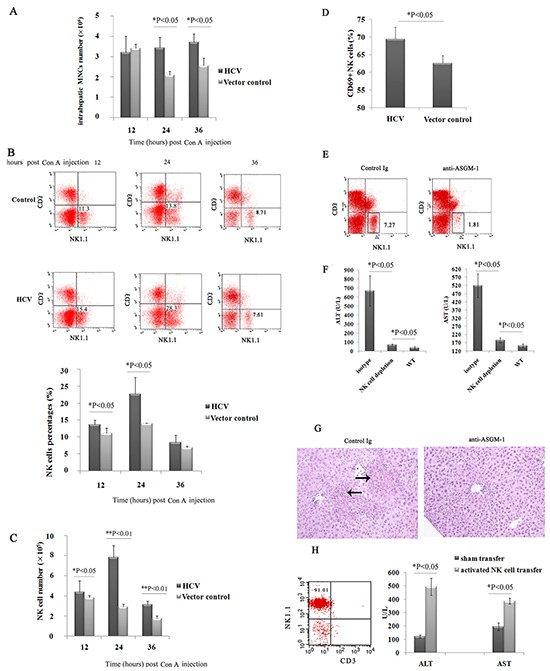
The increased liver injury induced by ConA was dependent on intrahepatic NK cells in HCV mice **A, B, C**. and **D**. Hepatic NK cell numbers and activation were upregulated in HCV mice after challenge with ConA. Mice injected with pattB-HCV-Fluc or pattB-Fluc were treated with ConA (10 μg/g body weight) and sacrificed at 12, 24 and 36 h after challenge. Hepatic MNCs were isolated and analyzed by flow cytometry using anti-NK1.1 and anti-CD3 antibodies. (A) The total number of intrahepatic MNCs is shown. The results represent one of three independent experiments (n=4). The percentages (B) and total numbers of (C) NK cells among hepatic MNCs are shown. (D) The surface expression of CD69 on CD3-NK1.1+ cells was also analyzed. *P<0.05. These results are representative examples from one of three experiments. (E, F and G) Depletion of NK cells alleviated ConA-induced hepatic injury. **E**. Depletion of NK cells was confirmed by flow cytometry. **F**. At 24 h after ConA treatment, serum ALT and AST levels were determined (n=5). *P<0.05. **G**. Representative photographs of H/E-stained liver sections obtained from an HCV mouse or an antibody-depleted HCV mouse at 24 h after challenge with ConA (magnification: 100×, the arrow indicates the parenchymal loss). **H**. Activated NK cells induced liver injury in NK cell-depleted HCV mice. Hepatic NK cells were isolated by negative selection using an NK cell isolation kit from HCV mice treated for 6 hours with ConA (10μg/g body weight). The purification of hepatic NK cells from 6-hour ConA-treated HCV mice is shown. Purified NK cells (1×10^6^) were adoptively transferred into the liver of anti-ASGM1-treated HCV mice that had been treated with ConA (10μg/g body weight) for 6 hours for the similar condition as the donor mice. Serum ALT and AST levels were measured 24 hours after NK cell transfer (n=3). Hepatic NK cells isolated from untreated HCV mice served as the control.

Because we confirmed that HCV expression in the murine liver decreases the hepatic NK cell population and leads to increased liver injury induced by ConA, we wondered whether the increased liver injury induced by ConA was dependent on intrahepatic NK cells in HCV mice. As shown in Figure 4A, after ConA injection, more intrahepatic MNCs accumulated in the HCV group than in the vector control group at 24 and 36 h (3.44×10^6^ versus 2.10×10^6^ at 24 h and 3.71×10^6^ versus 2.57×10^6^ at 36 h), indicating more severe hepatic inflammation in the HCV group. To characterize the subsets of hepatic lymphocytes, liver lymphocytes were separated and analyzed by three-color flow cytometry. Compared with the vector control-treated groups, both the proportion and number of NK cells in the HCV group were markedly increased at 12 h (13.75% versus 11.20%, 4.41×10^5^ versus 3.83×10^5^) and peaked at 24 h (22.80% versus 14.00%, 7.84×10^5^ versus 2.94×10^5^), persisting at 36 h post-ConA treatment (8.46% versus 6.91%, 3.14×10^5^ versus 1.77×10^5^, Figure 4B and 4C). The expression of CD69 on liver NK cells was increased at 24 h post-ConA treatment (69.37% versus 62.57%) in the HCV group (Figure 4D), reflecting the increased activation of these cells. No significant differences were observed in other lymphocyte populations, such as NKT or T cells, between the HCV and control mice, and hepatic NKT cells disappeared almost completely at 12 h after ConA-injection as previously reported by Hayashi et al. [18]. Depletion of the NK cells by pretreatment with anti-ASGM-1 mAb (Figure 4E) in HCV mice dramatically alleviated the ConA-induced hepatic injury, as indicated by significantly decreased serum ALT and AST levels (Figure 4F), and ameliorated histopathological changes (Figure 4G). This result was further confirmed by our observation that the adoptive transfer of activated hepatic NK cells from ConA-treated HCV mice could deliver liver injury in NK-depleted HCV mice (Figure 4H). However, *in vivo* depletion of NK cells by anti-ASGM1 administration did not inhibit ConA-induced liver injury in control mice, which is consistent with published findings (Supplementary Figure S8). Taken together, we conclude that NK cells play a crucial role in the hypersensitization of HCV mice to ConA-induced hepatic injury.

### IFN-γ, TNF-α, and perforin in liver NK cells contribute to increased liver injury in HCV mice

**Figure 5:**
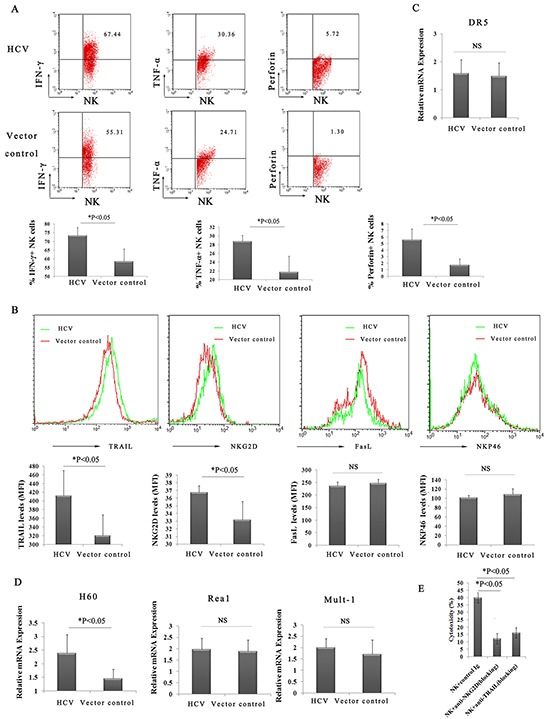
Highly activated hepatic NK cells and increased levels of cytokines act synergistically to amplify ConA-induced liver injury in HCV mice **A**. Enhanced production of IFN-γ, TNF-α and perforin from hepatic NK cells in HCV mice. Hepatic lymphocytes from p*att*B-HCV-Fluc-injected mice or p*att*B–Fluc-injected mice were prepared and examined by FACS using FITC-conjugated anti-NK1.1, PE-Cy^TM^5-conjugated anti-CD3e, PE-conjugated anti-IFN-γ, PE-conjugated anti-TNF-α, and PE-conjugated anti-perforin. The percentage of hepatic NK cells secreting IFN-γ, TNF-α and perforin at 24 h after ConA treatment is shown. The results represent the mean ±SD of triplicate samples. *P<0.05. **B**. Statistical analyses of the percentage of NKG2D+, TRAIL+, NKP46+, and FasL+ liver NK cells in HCV or control mice at 24 h after ConA treatment (n=5). *P<0.05. **C**. and **D**. Hepatocytes were prepared at 3 h after ConA injection for quantitative PCR analysis of DR5, H60, Rea 1 and Mult-1. The values shown represent the mean ± SD of six samples. *P<0.05. **E**. The 4-hour AST release assay was performed to assess the cytotoxicity of hepatic NK cells against hepatocytes. Hepatic NK cells purified from HCV mice treated for 2 hours with ConA (10μg/g body weight) were added to freshly isolated hepatocytes from HCV mice treated for 2 hours with ConA (10μg/g body weight) at an effector cell/target cell ratio (E/T) of 8/1. Hepatocytes (1×10^4^) were used as target cells. A dose of 20 μg/mL anti-NKG2D or anti-TRAIL (blocking) mAb was added to the culture for blockade.

The expression of IFN-γ, TNF-α, and perforin, which have all been implicated in the cytotoxicity of NK cells, was examined. Intracellular staining confirmed that the production of IFN-γ(73.34% versus 58.55%), TNF-α(28.77% versus 21.74%), and perforin (5.57% versus 1.73%) by liver NK cells increased at 24 h after ConA treatment (Figure 5A), suggesting an important involvement of IFN-γ, TNF-α, and perforin in HCV-associated liver injury.

### Enhanced TRAIL/DR5 and NKG2D/NKG2D ligand signaling pathways in HCV mice after ConA administration

To further determine the possible mechanisms underlying hepatocyte death, the expression of NK cell surface markers and their ligands on hepatocytes was investigated. As shown in Figure 5B, the expression of TRAIL and NKG2D was maintained at high levels on hepatic NK cells in the HCV group compared with the control group, whereas the expression of NKp46 and FasL was not upregulated at 24 h following ConA injection. The expression levels of H60, Rae-1 and Mult-1 (three important activating ligands of the NKG2D receptor) and death receptor 5 (DR5; also known as TRAIL-R2, KILLER, or TNFRSF10B) were also examined. Quantitative PCR revealed enhanced expression of H60 on hepatocytes in the HCV group compared with the control group (Figure 5D). In contrast, the expression of Rae-1, Mult-1 and DR5 exhibited no difference (Figure 5C and 5D). The role of TRAIL and NKG2D in the process was confirmed by direct cytotoxicity of the purified NK cells against hepatocytes from ConA-treated HCV mice in a 4-hour AST release assay *in vitro* (Figure 5E). Taken together, these results suggested that both the expression of TRAIL on hepatic NK cells and the NKG2D ligand (H60) in hepatocytes may play a crucial role in NK cell-mediated autoimmune liver injury in HCV mice, greatly activating hepatic NK cells via NKG2D/H60 and TRAIL/DR5 recognition.

## DISCUSSION

Susceptibility to liver injury represents a serious problem for the huge population of chronic HCV patients, as demonstrated by our ConA injection experiments in mice expressing HCV polyprotein. Although much has been learned in recent years, the pathogenetic mechanisms responsible for HCV-related liver disease progression remain poorly understood. It is thought that in human chronic hepatitis C, hepatocyte injury is not directly caused by HCV infection but is a consequence of the destruction of infected hepatocytes by cytotoxic lymphocytes. However, the latter requires a large amount of investigation, especially regarding the role of the hepatic NK cell compartment in the pathogenesis of HCV-associated hepatic failure.

Currently, most studies investigating the role of NK cells in HCV infection have focused on peripheral blood populations rather than on intrahepatic populations. Early studies have suggested that HCV inhibits NK cell functions and allows escape from the immune surveillance of NK cells, leading to chronic infection [28, 29]. Later, a prospective study was published that reaffirmed the importance of KIR2DL3-expressing NK cells in resistance to chronic HCV infection [30]. Multiple follow-up studies have suggested that peripheral NK cells are activated during chronic HCV infection and displayed an increase in cytotoxicity, with elevated expression of NKG2D, NKp46, and TRAIL [14, 31–34]. The elevated cytotoxicity of NK cells supported their potential contribution to liver injury.

Recent studies of NK cells in mouse and human livers have shown that NK cells are enriched in lymphocytes within the liver and have unique phenotypic features and functional properties compared with conventional NK (cNK) cells [14]. As a key component of innate immunity in the liver, NK cells perform critical roles in host defense against pathogens and tumors via their natural cytotoxicity and cytokine production, and they also act as regulatory cells by engaging in reciprocal interactions with other types of liver cells through cell-to-cell contact and the production of cytokines. Circumstantial evidence has suggested that the activation of hepatic NK cells represents a pivotal factor in pathogenetic processes, leading to progressive liver injury and ultimately to cirrhosis. It has been reported that hepatocellular damage and inflammation correlated with the activation of hepatic NK cells in patients with chronic HBV infection [14, 35]. However, there is still some discrepancy in intrahepatic NK cell activity in chronic HCV infection. Several studies investigating chronic HCV infections have emphasized differences between the intrahepatic NK cells and peripheral blood compartments, which showed that intrahepatic NK cells have significantly higher levels of TRAIL, NKp46, and CD122 expression and cytotoxicity than peripheral blood NK cells of HCV patients [31, 32]. However, this phenomenon was not confirmed in a subsequent study that failed to detect an increase in intrahepatic NK cell functions but instead showed a decrease in cytotoxicity and TRAIL expression in patients with chronic HCV infection [36]. Thus, the precise role of hepatic NK cells in liver injury during HCV infection remains obscure.

In this study, we found that the numbers of NK cells in the liver decreased markedly in the HCV group compared with the control group. Previously, significant depletion of NK cells has been reported in HCV-infected patients [9–12]. Our results confirmed the clinical data and showed that the forced expression of HCV proteins in murine livers without accompanying HCV replication could also result in the depletion of hepatic NK cells. Thus, although the precise mechanism leading to the inhibition of hepatic NK cells in mice integrated with HCV remains uncertain, it is likely that either the non-structural proteins of HCV, or the full complement of the viral proteins, are necessary for the observed immunomodulatory effect.

We also discovered a critical role of NK cells in oversensitive liver injury after triggering ConA in HCV mice. As immunomodulators, ConA was used to investigate the innate immunologic mechanisms of acute liver injury. We found that the expression of HCV in the liver potentiated ConA-induced liver injury, and we verified that the increased susceptibility correlated with hepatic NK cells. We observed that the significantly increased recruitment of NK cells into the liver, accompanied by the markedly increased expression of CD69 on hepatic NK cells following ConA treatment, closely correlated with the severity of hepatocyte injury, as reflected by increased serum levels of ALT and AST, as well as pathological changes in the HCV group. Furthermore, the depletion of NK cells in mice with integrated HCV dramatically alleviated the ConA-induced liver injury.

Interestingly, despite the clear protection elicited by NK cell depletion, a significant difference was also observed between the HCV groups treated with anti-ASGM-1 mAb and the normal mice (Figure 4F). These findings suggested that other cells might also contribute to liver injury after ConA injection in mice with integrated HCV. However, additional studies are needed to clarify this point. It has been shown that the activation of innate and adaptive immune cells, including natural killer T (NKT) cells, Kupffer cells, and CD4^+^ T cells, play essential roles in the pathogenesis of ConA-induced liver injury [18], while liver NK cell activation does not critically contribute to this phenomenon [37]. Our results showed that *in vivo* depletion of NK cells by anti-ASGM-1 administration did not inhibit ConA-induced liver injury in HCV-negative mice, which is consistent with the published findings (Supplementary Figure S6).

Recent studies have highlighted important roles of NK cells and their activating receptors in liver damage. Liver NK cells mediate hepatocyte toxicity in MHV-3-induced FHF both through Fas/FasL and NKG2D/NKG2DL pathways [38], kill freshly isolated hepatocytes by a TRAIL/TRAIL receptor pathway in polyinosinic-polycytidylic acid-induced mouse liver injury [39], and induce severe liver injury through NKG2D/NKG2D ligand (NKG2DL) recognition after low-dose ConA stimulation in HBV transgenic mice [19]. Here, we report the increased expression of NKG2D and TRAIL receptors as an additional strategy to enhance HCV-associated hepatic failure. In addition, the expression of NKG2D ligands (H60), which are known to be “stress-inducible” molecules that can be triggered by transformation or infection with viral and bacterial pathogens [40], was markedly enhanced following ConA stimulation in HCV mice. The increased expression of H60 on target cells may induce NK-mediated autoimmune responses. Additionally, we observed markedly increased production of perforin, IFN-γ and TNF-α by hepatic NK cells. Taken together, these findings suggest that highly activated hepatic NK cells and increased levels of cytokines act synergistically to amplify ConA-induced liver injury in HCV mice. Our results are at variance with data suggesting that HCV envelope or NS5A proteins impair NK cell activation and function, and they are consistent with recently published findings indicating an increase in intrahepatic NK cell functions in chronic HCV patients. This apparent discrepancy is likely due to the use of different models in these studies.

It has been reported that the entirepopulation of basophils constitutively expresses ASGM1 as well as CD49b (DX5), as does the NK cell population, and NK cell-depleting anti-ASGM1 antibody exhibits a lethal off-target effect on basophils *in vivo* [41]. Thus, we could not exclude the possibility that basophils might also contribute to the increased liver injury observed in HCV mice in addition to NK cells.

This study generated a new HCV mouse model as an interesting tool for the study of the immune-mediated mechanisms underlying HCV. The natural species tropism of HCV is limited to humans and chimpanzees. Although the chimpanzee model yielded valuable insight, a limited availability, high cost and ethical considerations limit their utility. Xenotransplantation and genetically humanized HCV mouse models are the only small animal models that can be infected with HCV, which had a major impact on the field of HCV research. However, these mice are highly immunodeficient, making studies of immunology and immunopathology impossible in these mice. Because the expression of the HCV viral polyprotein is an essential early step in the HCV life cycle, transgenic mice that conditionally express the full-length HCV ORF or a single recombinant protein have been reported, providing reasonable models to study the pathogenesis of this disease. However, the current models remain limited. For example, there is no immune response against the transgenic proteins in these mice, making it difficult to study the direct effects of HCV proteins on host intrahepatic immune and inflammatory responses. To overcome these limitations, we established a new mouse model for HCV by taking advantage of the liver-targeting manner of hydrodynamic injection, which has become well accepted as a potentially useful approach for the generation of novel mouse models for liver disease research, such as hepatitis B virus infection [42]. What is unique about this model is its immunocompetent features. These immunocompetent characteristics allow us to gain insight into the mechanism by which HCV induces hepatic injury in an immunologically naive small animal host. It should be stated that HCV proteins are expressed from a cellular promoter and that the untranslated regions are not included in the HCV plasmid. Consequently, no HCV replication is expected to occur in these cells, despite the production of the entire HCV coding region. Another distinct feature of this model is the ability to monitor HCV expression over time within individual animals by bioluminescence imaging of the luciferase reporter. The results in this article demonstrate that firefly luciferase is an accurate reporter for HCV expression in naive animals and is supported by a strong correlation between luminescence and HCV expression. This technique is relatively simple, robust, and extremely sensitive due to its exceptionally high signal-to-noise ratio, and it dramatically reduces the number of animals needed to generate statistically significant data [43].

This study generated an HCV mouse model as an interesting tool that can provide insight into HCV-induced liver pathophysiology and uncovered an essential mechanism by which hepatic NK cells contribute to the pathogenesis of HCV-induced liver injury.

## MATERIALS AND METHODS

### Mice

Specific pathogen-free (SPF) 6-week-old male C57BL/6 mice were obtained from the National Beijing Center for Drug Safety Evaluation and Research (NBCDSER). The mice were housed under SPF conditions. All animals received human care, and *in vivo* experiments were approved by the ethics committee of the NBCDSER (Permit No.10-823).

### Reagents

D-Luciferin was purchased from Promega (Madison, Wisconsin). Concanavalin A (type IV) (ConA) was obtained from Sigma-Aldrich Chemical Co (St. Louis, MO). Anti-mouse-TNF-α-PE, anti-mouse-IFN-γ-PE, anti-mouse-CD335(NKp46)-PE, anti-mouse-TRAIL-PE, anti-mouse-CD178(FasL)-PE, and anti-mouse-perforin-PE were obtained from eBioscience (San Diego, CA). Anti-mouse-CD134-PE, anti-mouse-CD69-PE, anti-mouse-IFN-γ-FITC, anti-mouse-CD11b-PE, anti-mouse-NK1.1-FITC, anti-mouse-CD11c-PE, and anti-mouse-CD3e-PE-Cy^TM^5 were obtained from BD Biosciences (San Jose, California).

### Plasmid construction and injection

The bicistronic plasmid p*att*B-HCV-Fluc was constructed using the human alpha-antitrypsin (AAT) enhancer/promoter region plus the coding region of firefly luciferase (Fluc), the internal ribosome entry site (IRES) of the encephalomyocarditis virus (ECMV) and the entire coding sequence of the HCV polyprotein. The AAT enhancer/promoter generated by amplification from the pBC-hFIX-B plasmid was inserted between the *Bgl*II and *I-pp*oI sites to replace the cytomegalovirus (CMV) promoter of the pCIneo plasmid (Promega, Madison, WI) to produce pA. The DNA fragment of *att*B was amplified using pT-*att*B [22] as a template and was inserted before the AAT enhancer/promoter at the *Bgl*II site to produce the pAA plasmid. The firefly luciferase gene reporter region was subcloned from the pGL3-Basic plasmid (Promega) into the HCV replicon of the genotype 1b pConI-FL vector [44] at the *Xba*I/*Dra*I site, digested with *Xba*I and *Eco*RV(approx. 11.8 kb) and inserted into the pAA vector to produce p*att*B-HCV-Fluc (Supplementary Figure S1). The p*att*B-Fluc plasmid that contained the *att*B site and firefly luciferase reporter gene controlled by the promoter region of AAT was constructed as a control (Supplementary Figure S1). The mouse-codon-optimized φC31 integrase-expressing vector Pphic31φ was obtained from Addgene, Cambridge, USA.

Plasmid DNA was purified using an Endotoxin Free Maxi Kit (Qiagen, Hilden, Germany). Plasmid DNA was administered to mice using a hydrodynamic-based gene transfer technique that was previously described by Liu et al [45]. Briefly, different amounts of plasmid DNA (10 to 30μg) in saline equivalent to 10% of the body weight (i.e., 2 ml for a 20 g mouse) were injected rapidly into the tail vein in less than 5–8 s using a 27-gauge needle.

### Cell isolation

Liver monocytes (MNCs) were prepared as described by Wang et al. [46]. Briefly, the liver was pressed through a 200-gauge stainless steel mesh and then suspended in RPMI 1640 (GibcoBRL, Gaithersburg, MD) medium containing 5% fetal bovine serum (Gibco BRL). After centrifugation at 1500 rpm for 10 min, the pelleted cells were resuspended in 40% Percoll (GE Healthcare, Uppsala, Sweden) solution, and the cell mixture was gently overlaid onto a 70% Percoll solution and further centrifuged at 2400 rpm for 20 min. The interface cells between the Percoll solutions were aspirated, washed twice with RPMI 1640 medium and centrifuged at 2000 rpm for 20 min at room temperature. The pellet was re-suspended in red blood cell lysis solution (155 mM NH_4_Cl, 10 mM KHCO_3_, 1 mM EDTA, and 170 mM Tris, pH 7.3) and then washed twice in 10% FBS- RPMI 1640 medium. The liver MNCs were counted by microscopy using a Burker-Turk prepared slide, and Wright-Giemsa staining was performed.

NK cells were isolated by negative selection using an NK cell isolation kit (STEM CELL technologies). The purity of the isolated fraction was measured by flow cytometry after labeling with CD3e-FITC and NK1.1-PE antibodies, yielding purities >89%.

To isolate mouse hepatocytes [19], the mice were anesthetized with sodium pentobarbital (intraperitoneally at30 mg/kg body weight), and then the portal vein was cannulated. The liver was subsequently perfused with ethylene glycol tetraacetic acid (EGTA) solution (5.4 mM KCl, 0.44 mM KH_2_PO_4_, 140 mM NaCl, 0.34 mM Na_2_HPO_4_, 0.5 mM EGTA, and 25 mM tricine, pH 7.2) and digested with 0.075% collagenase solution. The viable hepatocytes were then suspended in Dulbecco's Modified Eagle's Medium (Life Technologies, Gaithersburg, MD) and separated using 40% Percoll (Gibco BRL) with centrifugation at 420g for 10 minutes at 4°C.

### Cytotoxicity assay

To assay the cytotoxicity of hepatic NK cells against hepatocytes, a 4-hour AST release assay was performed [19]. Hepatic NK cells purified from 2-hour ConA-treated HCV mice were added to the freshly isolated hepatocytes from 2-hour ConA-treated HCV mice at the indicated effector/target (E/T) cell ratios. Hepatocytes (1×10^4^) were used as target cells in the assay. After 4 hours, the supernatant was harvested, and AST activity was measured. The specific cytotoxicity was calculated as [(AST_experimental_-AST_spontaneous_)/(AST_maximum_-AST_spontaneous_)]×100%.

### Cell depletion

Anti-ASGM-1 antibody was obtained from Wako Pure Chemical (Tokyo, Japan). A dose of 50 μg anti-ASGM-1 antibody was injected intravenously into the mice at 24 h prior to treatment. The elimination of NK cells was confirmed by flow cytometry.

### Bioluminescence imaging

Bioluminescence imaging was performed using an IVIS imaging system (Xenogen, Alameda, CA). The mice were *i.p*. injected with 150 mg/kg of D-luciferin. After 10 min, the mice were anesthetized using 1–3% isoflurane and imaged for luciferase expression. The regions of interest from representative images were quantified as photons/s/cm^2^/sr using Living Image software 4.2 (Xenogen).

### Flow cytometric analysis

Cells were stained with the indicated fluorescently labeled mAbs for surface antigens according to a standard protocol. For intracellular cytokine staining, after staining for extracellular markers, the cells were fixed in 1ml Fix/Perm buffer (eBioscience) for 60min at 4°C. After incubation in permeabilization buffer (eBioscience), the cells were stained with mAbs for intracellular cytokine antigens. The stained cells were analyzed using a flow cytometer (FACScalibur, BD, Franklin Lakes, NJ), and the resulting data were analyzed using Cell Quest Pro software.

### Histological and immunohistochemical evaluation

Livers were fixed in 10% neutral-buffered formalin for 24 h and then embedded in paraffin. The sections (5 μm) were affixed to slides, deparaffinized and subjected to hematoxylin/eosin (HE) or terminal deoxynucleotidyltransferase–mediated deoxyuridinetriphosphate nick-end labeling (TUNEL). Immunohistochemical staining using an anti-NS3 antibody (Millipore, Billerica, MA) and an anti-NS4 antibody (Abcam, Cambridge, United Kingdom) was performed to determine the expression rate of HCV proteins in the murine liver.

### Serum biochemistry

Serum biochemistry was measured using a TBA-200FR automated clinical chemistry analyzer (Toshiba, Tokyo, Japan).

### RNA extraction and RT-PCR

Total RNA was isolated from frozen liver samples collected at the indicated time points using TRIzol reagent (Invitrogen, Carlsbad, CA). First-strand cDNA was synthesized from 1 μg of total RNA using Rever Tra Ace (TOYOBO, Osaka, Japan), a reverse transcriptase, using random primers. Oligonucleotides used for PCR amplification of the luciferase, core, and GAPDH genes were as follows: luciferase forward, 5′-GAATTCATGGAAGACGCCAAAA-3′, reverse, 5′-GC GGCCGCTTACACGGCGATCTTTC-3′; Core forward, 5′-GCTCAATGCCTGAGATTGG-3′, reverse, 5′-TCATT GCCATAGAGAGGCCAA-3′; and GAPDH forward, 5′-ACCACAGTCCATGCCATCAC-3′, reverse,5′-TCCAC CACCCTGTTGC TGTA-3′.

### Quantitative PCR

RNA extraction and cDNA synthesis were performed using similar protocols as described for the RT-PCR assay. Quantitative PCR was performed using a Step one plus Real-Time PCR System (Applied Biosystems, Grand Island, NY) and the SYBR Green Real-Time PCR Master Mix (TOYOBO), according to the manufacturer's protocols. The primer sequences were as follows: GAPDH sense, 5′-ACCCAGAAGACTGTGGATGG-3′, antisense, 5′-ACACATTGGGGGTAGGAACA-3′; DR-5 sense, 5′-TGCTGCTCAAGTGGCGC-3′, antisense, 5′-GG CATCCAGCAGATGGTTG-3′; Rae1 sense, 5′-GC TGTTGCCACAGTCACATC-3′, antisense, 5′-CCTGG GTCACCTGAAGTCAT-3′; Mult1 sense, 5′-CAATG TCTCTGTCCTCGGAA-3′, antisense, 5′-CTGAACA CGTCTCAGGCACT-3′; and H60 sense, 5′-GTGTG ATGACGATTTGTTGAG-3′, antisense, 5′-ATTGATGG ATTCTGGGCCATC-3′. For analysis, the expression levels of the target genes were normalized to the housekeeping gene, GAPDH (ΔCt). The gene expression values were then calculated based on the ΔΔCt method as previously described [20]. Relative quantities (RQs) were determined using the equation RQ = 2^−ΔΔCt^.

### Analysis of genomic integration by nested PCR

Mice were sacrificed at 30 days after transfection, and the liver tissues were excised. Mouse liver genomic DNA was extracted using a Wizard Genomic DNA Purification Kit (Promega). Nested PCR was performed to identify hotspot integration sites. Mouse liver genomic DNA was used as a template for the first round of PCR with primers for *mps*L1 (forward), mspL1 (reverse) and *att*B-1. The products were used as templates in the second round of PCR with primers for *mps*L1 (forward), *msp*L1 (reverse) and *att*B-2. The second round of PCR products were cloned into pGEM-T and sequenced. The sequencing results were blasted against GenBank data. The sequences of the primers were *msp*L1 (forward) 5′-GTGGCACATTCCTTAATCCC-3′, *msp*L1 (reverse) 5′-TGAGGAGGAGCCTTAGCAAC-3′, *att*B-1 5′-GTA GGTCACGGTCTCGAAGC-3′ and *att*B-2 5′-CG AAGCCGCGGTGCGGGTGCCA-3′.

### Western blot analysis

For western blot analysis, liver cells were lysed in cell lysis buffer (Beijing CoWin Biotech, China) supplemented with 1 mM PMSF for 30 min on ice and then centrifuged at 12,000 g for 15 min at 4°C. The supernatants were mixed in loading buffer, boiled for 5 min, and then subjected to SDS–PAGE. After electrophoresis, the proteins were transferred onto PVDF membranes (Millipore) and probed with primary Abs overnight at 4°C. The membranes were washed with 0.1% (vol/vol) Tween 20 in TBS (pH 7.6) and incubated with a 1:5000 dilution of horseradish peroxidase-conjugated secondary Abs for 60 min at room temperature. Protein bands were visualized using the ECL reaction (Millipore). The expression of HCV genes was evaluated using anti-HCV Core mAb (1:1000 dilution; Abcam) and NS3 mAb (1:1000 dilution; Santa Cruz Biotechnology). Luciferase expression was evaluated using an anti-luciferase pAb (1:1000 dilution; Promega). An anti-GAPDH mAb (1:2000 dilution; Beijing CoWin Biotech) was used as an internal control.

### Statistical analysis

All experiments were performed at least twice, with a minimum of three replicates for each experiment. The data shown represent the mean ± SD. Statistical significance was determined using the two-tailed unpaired Student's *t*-test when two groups were compared. A probability value of <0.05 was considered statistically significant.

## SUPPLEMENTARY MATERIALS FIGURES


