# Supplementary material for: Hepatic NK cell-mediated hypersensitivity to ConA-induced liver injury in mouse liver expressing hepatitis C virus polyprotein
Source: Oncotarget. 2016 Aug 4;8(32):52178–92. doi: 10.18632/oncotarget.11052 (PMC5581021; doi:10.18632/oncotarget.11052)
Supplement: Supplementary file 1 [file oncotarget-08-52178-s001.pdf]

Hepatic NK cell-mediated hypersensitivity to ConA-induced liver injury in mouse liver expressing hepatitis C virus polyprotein

SUPPLEMENTARY FIGURES

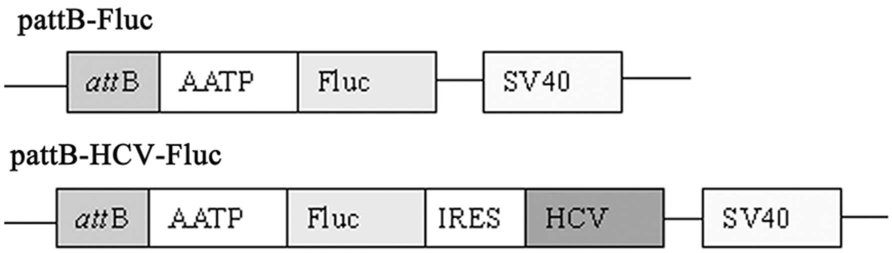

Supplementary Figure S1: A schematic representation of the plasmid used in this study. AATP, human alpha-antitrypsin promoter; *att*B, phage φC31integrase recognition site; Fluc, the firefly luciferase coding sequence; SV40, SV40 virus late polyadenylation signal sequence; HCV, Hepatitis C virus full-length coding sequence; EMCV IRES, internal ribosome entry site (IRES) of the encephalomyocarditis virus (ECMV).

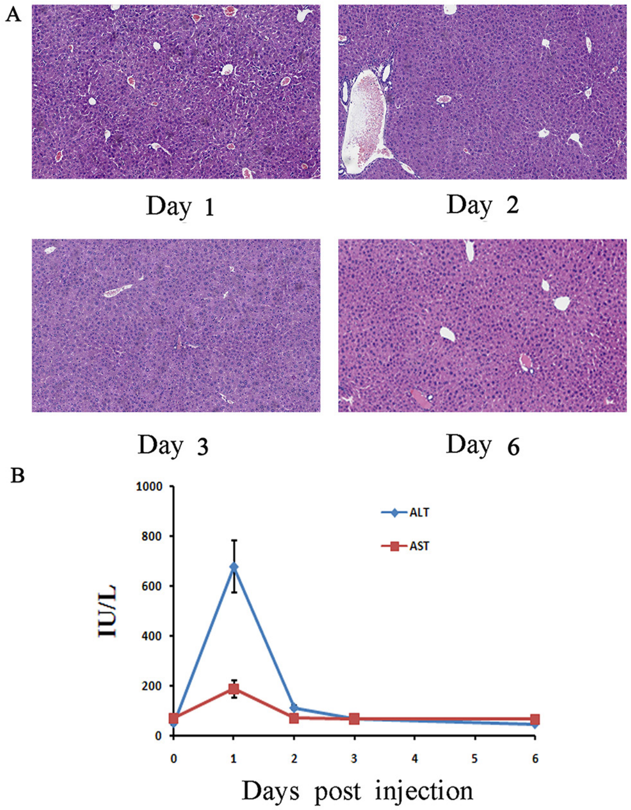

Supplementary Figure S2: The transient effect of HCV expression on mouse liver pathology. A. Histological examination of tissue damage through HE staining of liver sections from mice at 1, 2, 3, and 6 days after the hydrodynamic-based gene delivery. The results are representative of 3 experiments. Original magnification ×200. **B.** Serum ALT and AST levels at the indicated time points are shown. n=5 in each group.

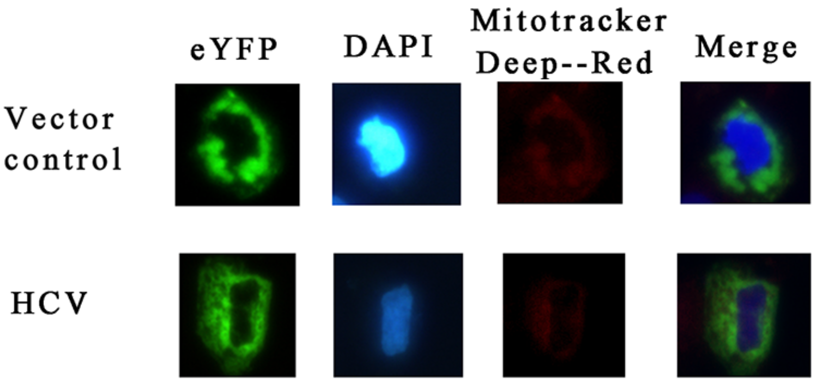

Supplementary Figure S3: Fluorescence microscopy of Huh7 cells cotransfected with eYFP-MAVS and p*att*B-HCV-Fluc at 48h post-transfection. Mitochondria were stained with Mitotracker deep red (red), and nuclei were labeled with DAPI (blue). Yellow labeling in the merged image indicates colocalization of eYFP-MAVS with mitochondria.

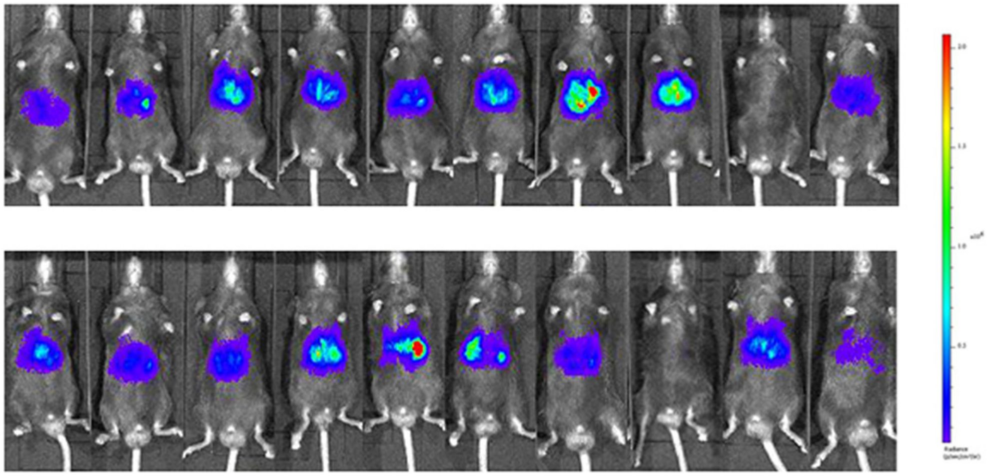

Supplementary Figure S4: The success rate of the hydrodynamic injection of the p*att*B-HCV-Fluc was monitored by bioluminescence imaging of the injected mice at 1 day after the injection, with 18 out of 20 injected mice exhibiting the expected luciferase expression within the liver. In another confirmation study, all 20 injected mice exhibited luciferase expression in the liver on day 1.

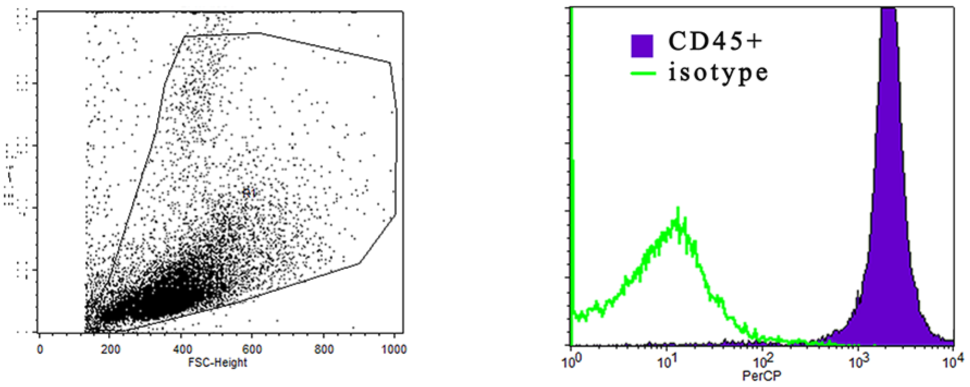

Supplementary Figure S5: FACS analysis illustrating that almost all interface cells between the Percoll solutions were positive for CD45.

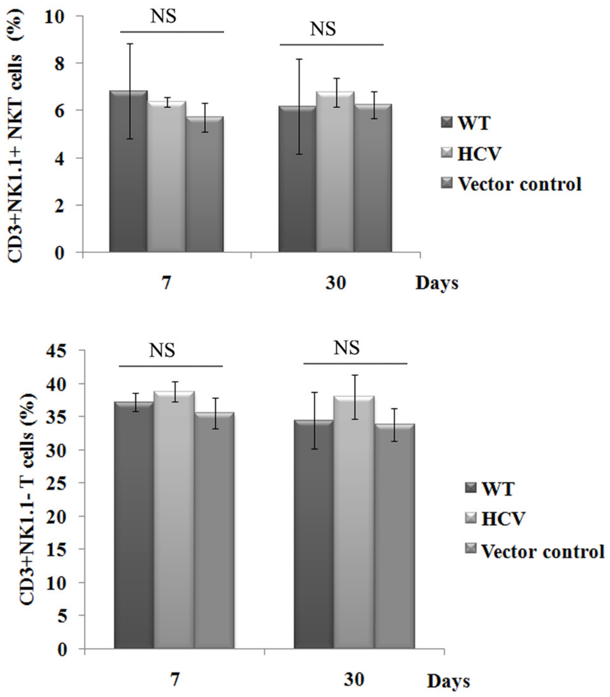

Supplementary Figure S6: Absolute numbers of CD3+NK1.1- T cells and CD3+NK1.1+ NKT were calculated by multiplying their respective percentages by the total number of liver MNCs.

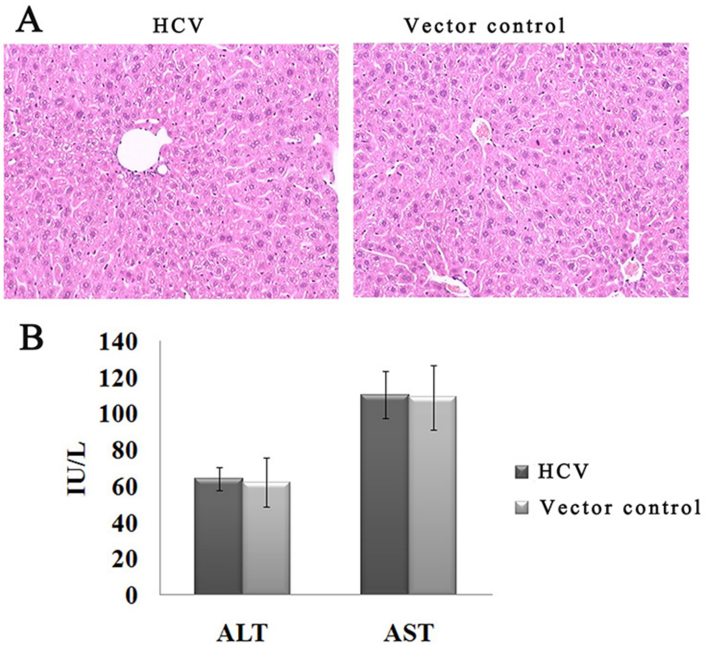

Supplementary Figure S7: Histological examination of liver sections (×100) **A.** and serum ALT and AST levels at 90 day post-integration (n=5) **B.**

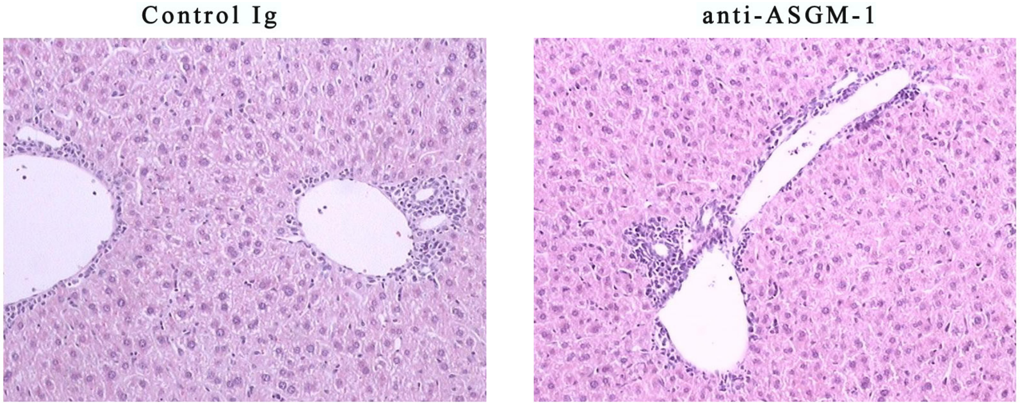

Supplementary Figure S8: Representative photographs of H/E-stained liver sections obtained from a control mouse or an antibody-depleted control mouse at 24 h after challenge with ConA (magnification: 100×).
